# Bullying Victimization and Quality of Life among Chinese Adolescents: An Integrative Analysis of Internet Addiction and Social Withdrawal

**DOI:** 10.3390/ijerph192416973

**Published:** 2022-12-17

**Authors:** Ning Ding, Xinwen Zhang

**Affiliations:** 1 School of Social and Behavioral Sciences, Nanjing University, Nanjing 210023, China; 2 Department of Social Work, The Chinese University of Hong Kong, Hong Kong, China

**Keywords:** bullying victimization, Internet addiction, social withdrawal, quality of life

## Abstract

Bullying victimization has been proven to be a direct predictor of adolescents’ quality of life, whereas elaborate mechanisms remain inconclusive. This study aims to explore the mediating pathway of Internet addiction and social withdrawal on the relationship between bullying victimization and quality of life among Chinese adolescents. This study used the cross-sectional data collected by self-reported questionnaires, including multidimensional peer-victimization scale, youth quality of life instrument-short form, prolonged social withdrawal questionnaire, and compulsive Internet use scale. A total of 1278 participants from four junior middle schools and two high schools participated in the questionnaire survey. SPSS 25.0 and Amos 25.0 were adopted to analyze the data. The results indicated that bullying victimization was directly and indirectly associated with adolescents’ quality of life. Internet addiction and social withdrawal partially mediated the relationship between bullying victimization and quality of life among adolescents. The current study demonstrated the underlying pathway of how bullying victimization affected adolescents’ quality of life, which could provide an intervention perspective for governments and social workers to improve adolescents’ quality of life by controlling Internet addiction and social withdrawal.

## 1. Introduction

Bullying victimization has been found as a common and severe international social issue [1]. Evidence from 28 western countries has reported that approximately 41% of students are victims of peer bullying [2]. Similar situations also have been found in mainland China. National research on a representative sample of students suggested that the prevalence of bullying victimization has reached 32.4% [3]. Bullying victimization refers to exposure to repeated and intentional aggressive behaviors, causing a power imbalance between perpetrators and victims [4]. As worrisome and traumatic experiences, bullying victimization is highly related to a series of adverse outcomes [5,6], such as depression [7], loneliness [8], and sleep disorders [9]. Poor quality of life (QoL) is also a detrimental consequence of bullying victimization [10]. As a comprehensive and broad indicator of adolescents’ well-being, QoL is commonly conceptualized as the individual perception of their position within their culture and value system, and in relation to their goals, expectations, standards, and concerns [11]. Negative associations between bullying victimization and high levels of QoL have been suggested in the literature [5]. This adverse impact gains greater severity when considering the importance of peer relationships in adolescence [12]. Nevertheless, the underlying mechanism of the effect of bullying victimization on adolescents’ QoL remains unclear.

Prior research has indicated that several latent factors play an imperative role in the correlation between bullying victimization and QoL among youths [13]. According to Reijntjes and his colleagues [7,14], these potential factors can be divided into externalizing and internalizing problems, which are caused by bullying victimization. For instance, the effect of bullying victimization on adolescents’ QoL may be mediated by internalizing symptoms, such as emotional problems [10]. In this study, Internet addiction is regarded as an indicator of externalizing symptoms, whereas social withdrawal is used as an indicator of internalizing symptoms [15]. Previous studies have demonstrated the negative effects of Internet addiction and social withdrawal among youths, which underscore further attention and intervention on them to improve adolescents’ well-being [16,17,18,19]. However, limited research has explored whether Internet addiction and social withdrawal mediate associations between bullying victimization and adolescents’ QoL in the Chinese context. 

Under these circumstances, this study aims to investigate the direct effect of bullying victimization on adolescents’ QoL, and the mediating roles of Internet addiction and social withdrawal. Findings may provide further precautionary and intervention measures for social work practice and the government to provide assistance to bullying victims.

## 2. Literature Review

### 2.1. Bullying Victimization and Quality of Life

The link between bullying victimization and adolescents’ QoL can be explained by trauma theory, which argues that, as the result of distressing and traumatic life events, trauma may weaken individuals’ normal and healthy self-image, leading to negative pathological effects [20]. From this viewpoint, bullying victimization is one of the incentives of trauma, which has a detrimental effect on adolescents’ daily life and decreases their QoL. The negative correlation between bullying victimization and adolescents’ QoL has been previously documented. Prior studies in European countries indicated that bullying victimization is a significant predictor of poor QoL among school-aged adolescents [12,21]. Similarly, despite considering the tolerance of bullying in different cultural contexts, a sample of Chinese students also demonstrated that school bullying victimization is negatively associated with adolescents’ QoL [22]. In addition, exposure to aggressive behaviors also has negative effects on specific measurement indicators of QoL. For instance, youths who have experienced bullying from their peers are at high risk of low life satisfaction, self-concept, and self-esteem, which may last into adulthood [23,24,25]. Therefore, bullied victims tend to experience more adverse outcomes and dysfunction, and their happiness with life remains permanently affected [26]. Compared with the non-disadvantaged, the disadvantaged perceive a sense of deprivation about individuals’ fundamental rights, which harms their psychological and physical development. Nevertheless, Lin and his colleagues [27] reached inconsistent conclusions, showing that the negative relationships between bullying victimization and adolescents’ QoL are indirect. The controversies on the effects of bullying victimization on adolescents’ QoL require research to elaborate on the relationship between bullying victimization and adolescents’ QoL.

### 2.2. Internet Addiction and Social Withdrawal as Mediators

Internet addiction, the most common externalizing symptom in adolescence, refers to the excessive and under-controlled use of online activities [28,29]. The self-medication hypothesis proposed that those who experienced stressful events tend to rely on medicine and substances to avoid adverse outcomes [30]. According to this hypothesis, Internet addiction can be regarded as a medicine, but it is an incorrect coping strategy and maladjustment of traumatic experiences [31]. That is, problematic Internet use may be caused by bullying victimization. When experiencing bullying victimization by their peers, individuals may immerse themselves in online activities to avoid repeated victimization and dealing with the adverse impact of being bullied [32]. Empirical research supported this viewpoint and indicated that bullying victimization contributes to online activity addiction, which may keep them away from distress and pain after exposure to stressful events [33]. Therefore, bullying victimization may positively predict adolescents’ Internet addiction.

However, the link between Internet addiction and adolescents’ QoL remains debatable. Previous studies indicated that Internet addiction is negatively associated with high QoL among adolescents as they escape from reality, which may reduce their interpersonal resources and developing opportunities [34,35]. Nevertheless, Tran and his colleagues [36] argued that being addicted to network activities allows adolescents to satisfy their psychological and social demands and may positively predict high QoL, which has been suggested empirically [37,38]. The discrepancy can be attributed to the different cultural norms. Consequently, although the negative influence of bullying victimization on Internet addiction has been proven, the correlation between Internet addiction and adolescents’ QoL in the Chinese context remains unclear.

Similarly, as one of internalizing symptoms, social withdrawal is defined as a state of voluntary social isolation [39], which manifests in three sub-types, namely, shyness, unsociability, and social avoidance [40]. Social withdrawal is highly relevant to peer relationships, which are significant for socialization after entering adolescence [41]. Bullying victimization, causing poor peer relationships that influence adolescents’ adjustment, is considered a predictor of social withdrawal. Bullying victims tend to withdraw from their peers to avoid aggressive behaviors and carry out rejection and passivity [39]. Recent research has mainly focused on the adverse outcomes and impact of social withdrawal [42,43], but has rarely explored the influencing mechanism of bullying victimization on adolescents’ withdrawn behaviors. 

Relevant research on the correlation between social withdrawal and adolescents’ QoL is also restricted. However, recent studies found that social withdrawal may affect individuals’ QoL-related factors. For instance, withdrawn adolescents engage in fewer social activities and reduce their social interactions, becoming more and more solitary and hopeless [44,45]. In addition to the influence of adolescents’ internal adaptation, social withdrawal is negatively associated with their social function in schools and families [46,47,48]. Scientific literature suggests that social withdrawal is detrimental not only to psychological maladjustment but also to the quality of social relationships that are essential to comprise individuals’ high QoL. Taken together, although limited research has focused on how social withdrawal directly influences adolescents’ QoL, increasing studies on the effect of social withdrawal on individuals’ internal and external adjustment provide reliable and relevant evidence. 

### 2.3. Present Study 

In conclusion, existing research emphasizes exploring the direct and indirect association between bullying victimization and adolescents’ QoL, whereas how bullying victimization influences adolescents’ QoL through other potential mechanisms remains inconclusive. Although bullying victimization is positively associated with Internet addiction, several controversies remain on the effect of Internet addiction on adolescents’ QoL. In addition, previous research on social withdrawal is limited, and whether this factor can be a mediator in the correlation between bullying victimization and adolescents’ QoL is unclear. With the aim to bridge these gaps, this study examines the underlying mechanisms of bullying victimization on QoL among adolescents in the Chinese context. Based on the theoretical framework and empirical evidence, three hypotheses are suggested as follows.

**H1:**  *Bullying victimization is negatively associated with QoL among adolescents*.

**H2:**  *Internet addiction would mediate the relationship between bullying victimization and adolescents’ QoL*.

**H3:**  *Social withdrawal would mediate the relationship between bullying victimization and adolescents’ QoL*.

## 3. Method

### 3.1. Participants

The data of this research was derived from a cross-sectional questionnaire investigation that was conducted in middle and high schools in Huai’an City, Jiangsu Province, from November to December 2021. The multi-stage cluster random sampling method was used to select students aged 12–18 years old from 4 junior middle schools and 2 high schools to participate in the questionnaire survey. Before the survey, we distributed the research prospectus to the invited students, informing them of the research content and the principles of anonymity and confidentiality. Subsequently, we issued students’ and guardians’ informed consents to 1454 students who were selected to participate in the questionnaire survey, and finally, we received informed consents from 1382 students and parents. With the guidance of trained research assistants, students who agreed to participate in the questionnaire survey completed the questionnaire independently in the classroom during their extracurricular activities, which took about 40 min. Research assistants examined the quality of questionnaires and reviewed them after students submitted questionnaires. In total, 104 questionnaires were found to have many missing contents through screening, so they were discarded, and the final number of valid questionnaires was 1278. All research materials in this study were reviewed and approved by the first author’s university research ethics committee.

### 3.2. Measures

#### 3.2.1. Demographics

Some demographic variables were collected in this study, including adolescents’ gender, age, grade, household registration (Hukou), and the education level of the parents.

#### 3.2.2. Predictors 

The predictor of this research is bullying victimization. The multidimensional peer-victimization scale [49] was used to measure bullying victimization experienced by adolescents in the past year. There are a total of 16 items on this scale (e.g., called me names; took something of mine without permission), mainly divided into four dimensions of physical victimization, verbal victimization, social manipulation, and attacks on property. The participants were asked to answer each item on a three-point scale ranging from 1 = never to 3 = more than once. The higher the score was, the more serious the adolescents’ bullying victimization was. The Chinese version of the scale proved to have good reliability and validity [50]. The Cronbach’s alpha of this scale was 0.909 in this study.

#### 3.2.3. Outcome Variables

Quality of life is the outcome variable in this research. The adolescents’ quality of life was measured by the youth quality of life instrument-short form (YQOL-SF) [51] in this study. A total of 15 items are included in this scale (e.g., feeling comfortable with the amount of stress in your life; feeling comfortable with your sexual feelings and behaviors), which comprised four dimensions: sense of self, social relationships, environment, and general quality of life. The participants were asked to answer each item on a five-point scale ranging from 1 = absolutely disagree to 5 = absolutely agree, and higher grades suggested higher quality of life among adolescents. The Chinese version of the scale was confirmed to have good reliability and validity [52]. The Cronbach’s alpha of this scale was 0.881 in this study.

#### 3.2.4. Potential Mediators

Social withdrawal is one of the potential mediators in this study. The prolonged social withdrawal questionnaire (HQ-25) [53] was used to measure the degree of adolescents’ social withdrawal. The scale has 25 items in total (e.g., I stay away from other people; I spend most of my time at home), mainly divided into three dimensions: socialization, isolation, and emotional support. The participants were asked to answer each item on a five-point scale ranging from 1 = absolutely disagree to 5 = absolutely agree, and higher scores indicated higher levels of social withdrawal. The good reliability and validity were proven in the Chinese version of the scale [54]. In the current study, the Cronbach’s alpha was 0.915.

Internet addiction is another potential mediator of this research. This study used the compulsive Internet use scale (CIUS) [55] to measure the severity of adolescents’ Internet addiction. This scale includes 14 items in total (e.g., do you find it difficult to stop using the Internet when you are online; do others say you should use the Internet less?), which were divided into five dimensions of loss of control, conflict, preoccupation, coping, and withdrawal symptoms. The participants were asked to answer each item on a five-point scale ranging from 1 = never to 5 = very often, and the higher the grade was, the more serious the Internet addiction was. The good reliability and validity were confirmed in the Chinese version of the scale [56]. In the current study, the Cronbach’s alpha was 0.804.

### 3.3. Analytical Plan

All analyses of this study were carried out using SPSS 25.0 and Amos 25.0. Firstly, we used SPSS 25.0 to analyze the basic statistical description of the demographic characteristics of the participants in this study, mainly calculating the gender, grade, household registration (Hukou), the frequency of parental education, as well as the mean and standard deviation of the participants’ ages. Second, we calculated the mean and standard deviation of four core variables (bullying victimization, quality of life, social withdrawal, and internet addiction), as well as their correlates. Third, we adopted Amos 25.0 software to carry out path analysis, mainly verifying the direct and indirect effects of bullying victimization on adolescents’ quality of life. The test for mediating effects of social withdrawal and internet addiction mainly adopted the bootstrap method with 5000 resamples and 95% bias-corrected confidence intervals for significant testing.

## 4. Results

### 4.1. Characteristics of Social Demographic Variables

Table 1 presented the characteristics of social demographic variables among adolescents. In the sample, the adolescents were between 12–18 years old (M = 15, SD = 1.65), of whom 780 (61%) were middle school students and 498 (39%) were high school students. 592 (46.3%) participants were male and 686 (53.7%) were female. 580 (45.4%) participants were born in rural areas and 698 (54.6%) participants were born in towns or cities. Besides, 57.7% of their fathers and 44.4% of their mothers were at a senior high school graduation level or above.

### 4.2. Correlations among the Main Variables

Table 2 presented the descriptive statistics and correlations among the main variables (bullying victimization, social withdrawal, internet addiction, and quality of life). The results suggested that bullying victimization was positively correlated with social withdrawal (r = 0.288, *p* < 0.01) and internet addiction (r = 0.156, *p* < 0.01), and was negatively related to adolescents’ quality of life (r = −0.317, *p* < 0.01). Meanwhile, social withdrawal (r = −0.680, *p* < 0.01) and internet addiction (r = −0.380, *p* < 0.01) were negatively associated with the quality of life of adolescents. A significant correlation was also found between social withdrawal and internet addiction (r = 0.419, *p* < 0.01). 

### 4.3. Testing of the Direct and Indirect Effects

**Figure 1:**
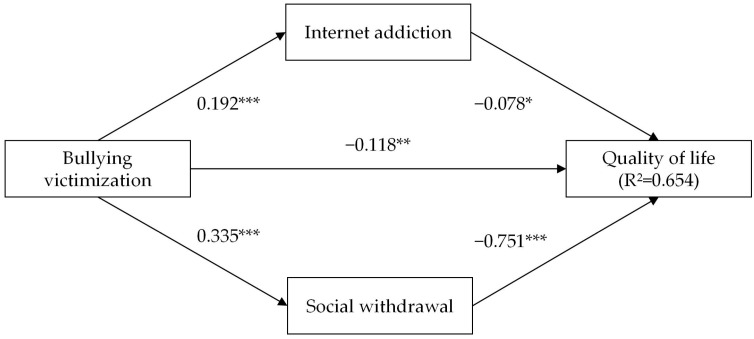
The mediating model (*** *p <* 0.001; ** *p <* 0.01; * *p <* 0.05).

The structural equation model with Amos 25.0 was adopted to test the direct and indirect effects. The results demonstrated that the model fits well with the data: χ^2^ = 371.02, df = 72, *p* < 0.001, CFI = 0.919, and RMSEA = 0.061. Figure 1 shows the effecting path of the mediating model. 

As shown in Figure 1 and Table 3, the results showed that bullying victimization had a negatively direct effect on adolescents’ quality of life (β = −0.118, *p* < 0.01), while bullying victimization had a positively direct effect on internet addiction (β = 0.192, *p* < 0.001) and social withdrawal (β = 0.335, *p* < 0.001). Besides, internet addiction (β = −0.078, *p* < 0.05) and social withdrawal (β = −0.751, *p* < 0.001) had a negative direct effect on the quality of life of adolescents. Thus, the effect of bullying victimization on adolescents’ quality of life was partially mediated by internet addiction and social withdrawal. All variables in the model accounted for 65.4% of the explained variance to quality of life (R^2^= 0.654).

In order to further verify the mediating effects of internet addiction and social withdrawal, the bootstrap estimation with 5000 resamples and 95% bias-corrected confidence intervals in Amos 25.0 were utilized in the current study. The standardized indirect effect and 95% CI were shown in Table 4. The results showed that the standardized indirect effect of bullying victimization on quality of life mediated by internet addiction was −0.061 (95% CI = [−0.105, −0.026], *p* < 0.01). The 95% confidence interval of the indirect effects did not contain zero, along with the significant direct effects, indicating that internet addiction partially mediated the relationship between bullying victimization and quality of life. Moreover, the results also demonstrated that the standardized indirect effect of bullying victimization on quality of life mediated by social withdrawal was −0.247 (95% CI = [−0.339, −0.162], *p* < 0.001). The 95% confidence interval of the indirect effects did not contain zero, along with the significant direct effects, indicating that social withdrawal could partially mediate the relationship between bullying victimization and quality of life.

## 5. Discussion

This study aimed to examine the relationship between bullying victimization and adolescents’ QoL, as well as the mediating role of Internet addiction and social withdrawal. As hypothesized, the results supported all our hypotheses, which reveal that bullying victimization is not only negatively associated with adolescents’ QoL, but also has an indirect impact on adolescents’ QoL through the mediating paths of Internet addiction and social withdrawal. The empirical evidence and findings are discussed as follows.

Findings verified H1 and suggested negative relationships between bullying victimization and adolescents’ QoL. The findings were consistent with trauma theory, which indicates that bullying victimization may cause trauma for adolescents and then induce a host of negative consequences related to QoL [20]. Most of the previous literature has reached similar conclusions, showing negative associations between bullying victimization and adolescents’ QoL [12]. Apparently, violent behaviors from peers tend to put bullying victims in a disadvantaged position and deprive them of the right to a normal life, resulting in a decline in QoL. However, these results were contrary to those of Lin and his colleagues [28], who found no direct association between bullying victimization and adolescents’ QoL. This difference showed that emotional problems play a fully mediating role in the correlation between bullying victimization and adolescents’ QoL. In conclusion, the present findings supported the application of trauma theory and demonstrated that bullying victimization can negatively influence adolescents’ QoL in the Chinese context.

Empirical findings also verified the second and third hypotheses, demonstrating that Internet addiction and social withdrawal mediate the link between bullying victimization and QoL among adolescents. First, bullying victimization was positively correlated with Internet addiction. This finding was in line with the self-medication hypothesis [30], which suggested that being addicted to online activities is an inappropriate coping strategy for bullying victims. Similar effects were found in previous studies. When individuals have difficulties in meeting their expectations in real life, they prefer to find satisfaction elsewhere [57]. In this sense, victims are unable to gain approval and acceptance from their peers, and thus may satisfy their psychological needs and gain their confidence by engaging in online activities. Second, the result showed that Internet addiction has negative effects on adolescents’ QoL, which was consistent with previous research demonstrating that those who indulge in online networks are vulnerable to daily life dysfunction [34]. However, these results were not aligned with conclusions about the positive effect of Internet addiction on adolescents’ QoL. These different findings may be induced by varying cultural norms as mentioned above. In summary, this study confirmed the mediating role of Internet addiction in correlations between bullying victimization and adolescents’ QoL. 

The mediating path of social withdrawal was also significant in this study. Aggressive acts may increase victims’ withdrawn behaviors, and further decrease opportunities to generate high QoL among adolescents in the Chinese context. The literature has focused on how social withdrawal affects adolescents’ psychological maladjustment or social relationships. As a supplement to this field, this study focused on adolescents’ QoL. First, the victimization experience can increase adolescents’ withdrawn tendencies. This finding was consistent with previous research that showed peer victimization and social withdrawal have positive correlations [18]. The possible reason is that threatening external environments may trigger adolescents’ defense mechanisms to maintain a positive self-image. Thus, for victims, social withdrawal is a self-defensive approach to coping with bullying behaviors. Second, socially withdrawn individuals are more likely to be trapped in troubles and hardships in their life. This can be explained by the fact that social withdrawal interferes with adolescents’ access to social interactions (including parents, peers, and teachers) and restricts their capacity to solve social problems [58,59]. Without social participation, withdrawn adolescents experience difficulties in cultivating the skills needed to cope with crises in social and psychological development, which in turn reduces the levels of their QoL [60]. Therefore, withdrawn youths may not only fail to learn skills from social interaction with others but may also be discriminated against by society, which further decreases their QoL in the Chinese cultural context. Taken together, these findings substantiated that social withdrawal mediates the correlation between bullying victimization and QoL among adolescents.

## 6. Implications

In summary, this study supplied empirical evidence for the negative link between bullying victimization and QoL among adolescents and the mediating effects of Internet addiction and social withdrawal in the Chinese cultural background. Theoretical contributions and practical implications that may promote the QoL of bullying victims are suggested below.

In terms of theoretical values, the feasibility of several theories, including trauma theory and the self-medication hypothesis on the associations among bullying victimization, Internet addiction, social withdrawal, and adolescents’ QoL, are examined and discussed with respect to the Chinese cultural background. The empirical evidence in this research supplements the cultural perspective of the two theories. This study also provides empirical support for the integrated theoretical framework in which bullying victimization has an indirect influence on Chinese adolescents’ QoL through the mediating mechanisms of Internet addiction and social withdrawal. This mediation model expands upon the current literature on direct relationships between bullying victimization and adolescents’ QoL, suggesting future research should focus on Internet addiction and social withdrawal. Moreover, this study concentrates on the mediating role of social withdrawal in the correlation between bullying victimization and adolescents’ QoL. The validity of mediating mechanisms of social withdrawal provides future studies with an analysis model, which indicates that social withdrawal mediates the negative correlation between stressful life events and their adverse outcomes. 

In terms of practical implications, given the adverse effects of bullying victimization on QoL among adolescents, precautions and intervention measures are necessary and require attention. At present, governments, schools, and communities should take precautionary measures against bullying victimization. First, governments should propose several regulations on the prevention of bullying victimization, such as dividing legal obligations on bullying for schools and teachers [61]. Second, as the most common place for bullying, schools can build a safe environment and comfortable school climate that may foster friendly teacher–student and peer relationships, which is also effective in curbing aggressive behaviors, as seen in the project “Steps to Respect” [62]. Third, communities may provide parent–child programs to cultivate positive parenting and enhance family communication. Such programs are conducive to identifying adolescents’ victimization and provide them with psychological support and coping, as seen in the Incredible Years Program. Furthermore, if bullying does occur, social workers can adopt art therapy to assist bullying victims in freely expressing their emotions and thoughts, which allows for reestablishing their positive self-perception after bullying victimization [63]. 

The crucial role of Internet addiction and social withdrawal in the influencing paths of bullying victimization on adolescents’ QoL emphasizes the need for further intervention for victims. First, given the negative effects of Internet addiction on adolescents’ QoL, the authorities must take measures to settle the issue of Internet addiction among adolescents. At present, the Chinese government has launched a game as an anti-addiction system, which limits the time for teenagers to engage in online games. Such measures effectively restrain their Internet addiction. In addition, considering that bullying may exacerbate Internet addiction, counseling services and psychological interventions must be provided by communities and schools to help disadvantaged groups wisely engage in the network and effectively cope with addiction [64]. Furthermore, social workers can establish alliances with disadvantaged adolescents through prolonged conversations, which can also provide them with a secure and private consulting basis [65]. This practice can contribute to understanding and mastering these teenagers’ past trauma and present psychological conditions [66]. Otherwise, group social work can provide withdrawn adolescents with a social health space. The program “free space” aims to encourage isolated youth to congregate and increase opportunities for interaction with others. Considering the nature of social withdrawal, social workers need to adjust the activity groups to the social orientation of adolescents to achieve the best therapeutic effect [65]. 

## 7. Limitations

Several limitations must be noted in this study. First, a cross-sectional sample was adopted but presented still inconclusive casual associations between bullying victimization, Internet addiction, social withdrawal, and adolescents’ QoL. Thus, future research may explore the causality among bullying victimization, social withdrawal, Internet addiction, and adolescents’ QoL through longitudinal designs. Second, this study only examined the effect of bullying victimization on adolescents’ QoL but did not explore different dimensions of victimization (physical, verbal, relational). Therefore, further differences between various forms of bullying victimization may be identified to provide more targeted interventions for adolescents in the future. Third, reporting and recall basis may exist in this research because of the nature of self-reporting data. Consequently, multiple methods of data collection on bullying victimization among adolescents, such as surveying their parents and teachers, can be encouraged for future studies. Finally, the study object was derived from only one province in mainland China. Whether the results can be applied to other regions and countries remains unclear. Hence, future studies must examine the applicability of this model in a different cultural context.

## 8. Conclusions

The results of this study denoted that bullying victimization has direct and indirect effects on the quality of life among adolescents, through the mediating role of social withdrawal and Internet addiction. From the perspective of public health, early identification and precaution on bullying victimization could promote higher levels of quality of life among adolescents. Furthermore, intervention in social withdrawal and Internet addiction should also be considered by adolescents’ healthcare and services. 

## Figures and Tables

**Table 1:** Characteristics of the participants.

Variables	N (%)/Mean (SD)
Gender	
Male	592 (46.3%)
Female	686 (53.7%)
Age	15 (1.65)
Grade	
Seventh	276 (21.6%)
Eighth	280 (21.9%)
Ninth	224 (17.5%)
Tenth	162 (12.7%)
Eleventh	176 (13.8%)
Twelfth	160 (12.5%)
Household registration	
Rural	580 (45.4%)
Urban	698 (54.6%)
Father’s education	
Below primary school	24 (1.9%)
Primary school graduation	86 (6.7%)
Junior high school graduation	430 (33.6%)
Senior high school graduation	572 (44.7%)
Junior college graduation	88 (6.9%)
College or higher	78 (6.1%)
Mother’s education	
Below primary school	66 (5.2%)
Primary school graduation	158 (12.4%)
Junior high school graduation	486 (38%)
Senior high school graduation	418 (32.7%)
Junior college graduation	104 (8.1%)
College or higher	46 (3.6%)

**Table 2:** Descriptive statistics and correlations among main variables.

Variable	Bullying Victimization	Social Withdrawal	Internet Addiction	Quality of Life
Bullying victimization	-			
Social withdrawal	0.288 **	-		
Internet addiction	0.156 **	0.419 **	-	
Quality of life	−0.317 **	−0.680 **	−0.380 **	-
Min	1	1	1	1
Max	3	5	4.65	4.60
Mean	1.45	3.73	2.40	2.42
Standard deviation	0.46	0.75	0.73	0.71

** *p <* 0.01.

**Table 3:** Coefficients of path analysis.

Predictors	Outcomes	β	*p*
Bullying victimization	Quality of life	−0.118	**
Bullying victimization	Internet addiction	0.192	***
Bullying victimization	Social withdrawal	0.335	***
Internet addiction	Quality of life	−0.078	*
Social withdrawal	Quality of life	−0.751	***

* *p <* 0.05; ** *p <* 0.01; *** *p <* 0.001.

**Table 4:** Standardized indirect effect and 95% CI.

Pathway	β	Lower	Upper
Bullying victimization→Internet addiction→Quality of life	−0.061 **	−0.105	−0.026
Bullying victimization→Social withdrawal→Quality of life	−0.247 ***	−0.339	−0.162

** *p <* 0.01; *** *p <* 0.001.

## Data Availability

The data presented in this study are available on request from the corresponding author.
